# Detection of *Cryptosporidium parvum* and *Cyclospora cayetanensis* infections among people living in a slum area in Kathmandu valley, Nepal

**DOI:** 10.1186/s13104-017-2779-2

**Published:** 2017-09-07

**Authors:** Balkrishna Bhattachan, Jeevan Bahadhur Sherchand, Sarmila Tandukar, Bhim Gopal Dhoubhadel, Leesa Gauchan, Ganesh Rai

**Affiliations:** 1 Shi-Gan International College of Science and Technology, Kathmandu, Nepal; 2 Siddhi Memorial Hospital, Bhimsenstan, P.O. Box 40, Bhaktapur, Nepal; 3 Department of Microbiology and Public Health Research Laboratory, Tribhuvan University Teaching Hospital, Kathmandu, Nepal; 4 School of Tropical Medicine and Global Health, Nagasaki University, Nagasaki, Japan; 5 Bhaktapur Hospital, Bhaktapur, Nepal

**Keywords:** *Cryptosporidium*, *Cyclospora*, Infections, Slum, Kathmandu, Nepal

## Abstract

**Objective:**

The aim of this study is to determine the prevalence of *Cyclospora cayetanensis* and *Cryptosporidium parvum* infections among people living a slum in Kathmandu valley, Nepal.

**Results:**

Ten different parasites were detected in the stool samples; the prevalence of any parasite was in 27.1% (71/262). The prevalence of *C. cayetanensis* and *C. parvum* were 14.1% (10/71) and 5.6% (4/71), respectively. This study showed high prevalence of intestinal parasitic infections along with the coccidian parasites in the slum area of Kathmandu Valley.

**Electronic supplementary material:**

The online version of this article (doi:10.1186/s13104-017-2779-2) contains supplementary material, which is available to authorized users.

## Introduction

*Cryptosporidium* and *Cyclospora* spp are obligate, intracellular, coccidian protozoan parasites that infest the gastrointestinal tract of humans and animals causing diarrhea illness. In the environment, both type of oocysts excreted in the feces of infected individuals [1]. *Cryptosporidium parvum* is an important causative agent of human and animal gastrointestinal illness globally [2]. Oocysts of *C. parvum* were detected in domestic wastewater from December 2006 to April 2007 in China [3]. It was also isolated from stool samples among children in 1998 and 2007 in India [4]. In Nepal, the first report of *C. parvum* was from a 3-year-old boy with chronic diarrhea in Kanti Children Hospital in Kathmandu in 2001 [5]. *Cryptosporidium* was detected as a common cause of acute diarrhea in children less than 5 years old [6].

*Cyclospora cayetanensis* infection has emerged as a cause of acute and chronic gastroenteritis worldwide [7]. It has been reported from various parts of the world from Southeast Asia to South America [8–12]. An outbreak of *Cyclospora* infection was reported among adults in November 2004 in Peru [13]. Source of water is probably an important vehicle, either drinking parasite contaminated water directly or when it is used to growing vegetables. Moreover, it has been implicated in outbreaks in various countries including both developed (e.g. the United States) and developing countries (e.g. Nepal) [14–16].

The prevalence of these coccidian parasites is not well characterized among slum-dwellers, who are marginalized people and considered to have low facility of safe water and hygiene. This study was carried out at a slum in Kathmandu valley, aiming to determine the prevalence of *C. parvum* and *C. cayetanensis* in the stools of people living in that area.

## Main text

### Methods

The descriptive and cross-sectional study was carried out at Thapathali slum area (2.4 km^2^) of Kathmandu valley in November 2013. About 1000 people were residing in that area. Stool samples were collected from at least one member of a household if available from all households of the slum area. The inclusion criteria were: those participant whom were ready to participate and provide stool, water and along with written consent; and the exclusion criteria were: those who did not want to participate in the study. Participant are grouped as literate if they had at least primary level of education or who could read or write; and as illiterate if they were unable to read or write.

Using a standard questionnaire demographic data were collected. Stool samples were collected from people in the slum area after giving proper instruction. From each person, stool samples (about 30 g or nearly 30 ml of fresh stool) were collected in a clean, dry and screw capped container, by avoiding contamination with urine, water and other substances. Stool samples were kept in icebox during collection time and by maintaining cold chain all the samples were transported to the Public Health Research Laboratory, Institute of Medicine, Kathmandu, Nepal within the same day for laboratory examination.

### Formal-ether sedimentation method

About 3 ml stool samples in test-tube was shaken well and filtered by using cotton gauge. Three milliliter of ether was added and shaken well and the liquid suspension was centrifuged. The sediment portion was examined by microscopy with adding saline solution. Cyst and trophozoite of parasites were detected by microscopically under 10× followed by 40× objectives [17]. Microscope Olympus CXX2 was used for the microscopy. All the positive stool samples were further confirmed by modified acid fast staining method.

### Modified acid fast staining method

**Fig. 1:**
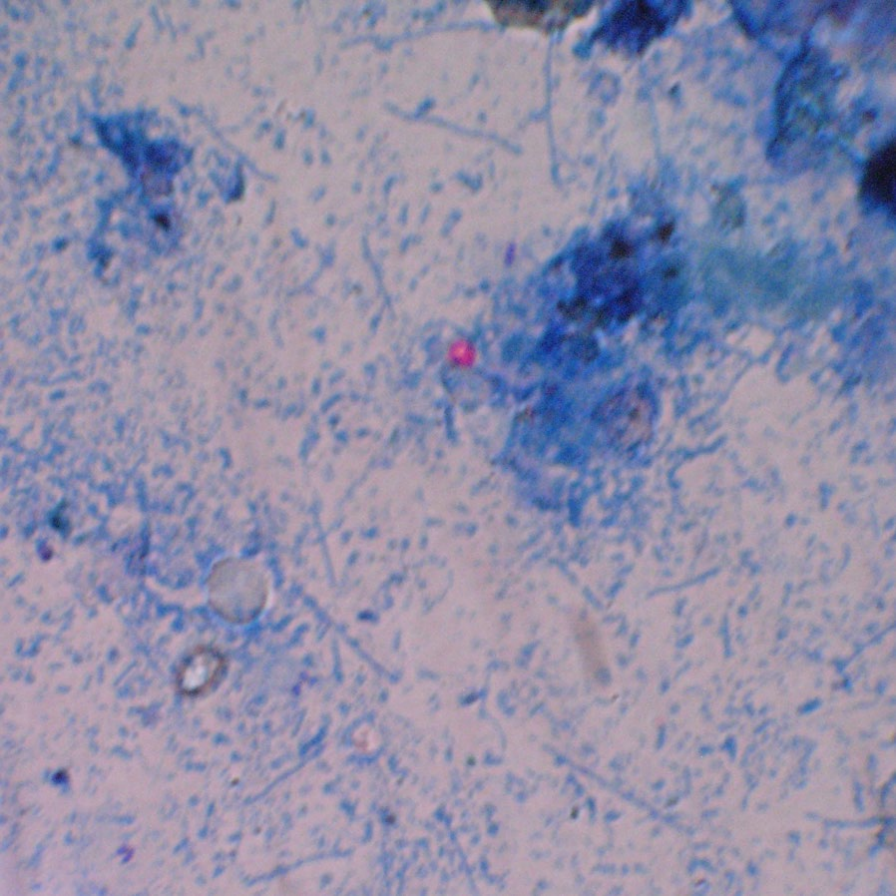
Photograph of oocyst of *Cryptosporidium parvum* (Ziehl–Neelsen staining at 100× magnification) found in this study

**Fig. 2:**
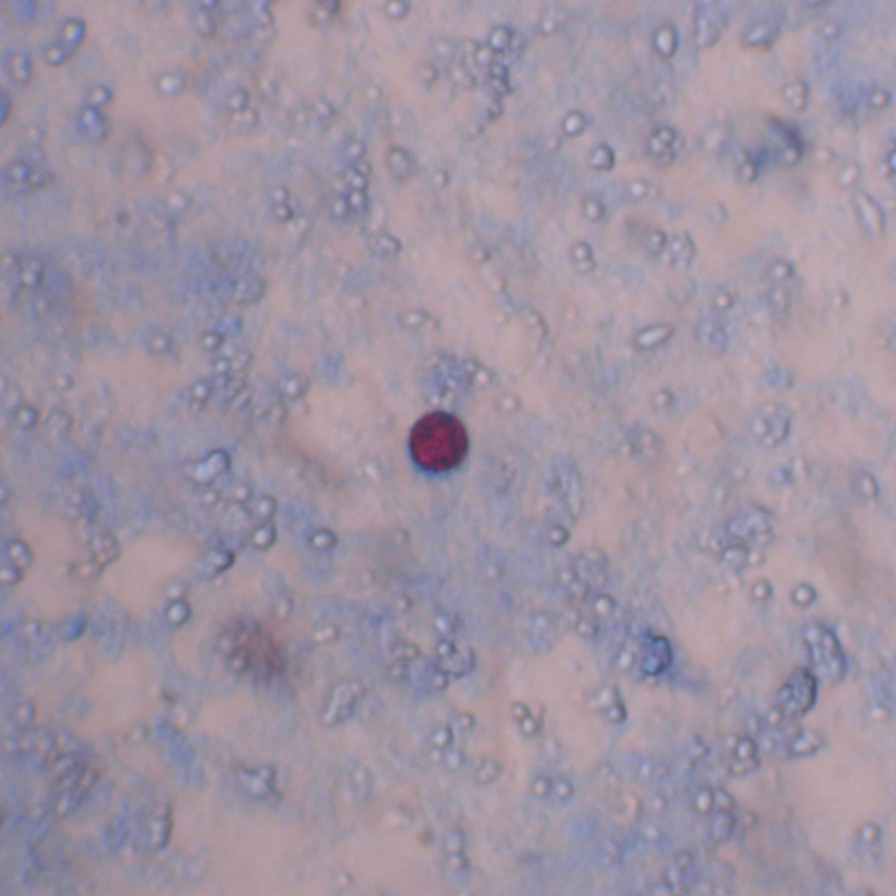
Photograph of oocyst of *Cyclospora cayetanensis* (Ziehl–Neelsen staining at 100× magnification) found in this study

Dry and fixed smears were prepared on dry glass slide by letting them dry on air and heating gently. The smears were flooded with carbol fuchsin (S005) till 8–10 min and heated to steaming for fixation. They were rinsed with tap water, decolorized with 5% aqueous sulfuric acid (S033) for 2 min or until film exhibited faint pink colour, and rinsed again. The smears were then flooded with methylene blue (S022) counterstain for 30 s, rinsed with tap water, drained, and air dried; and observed under oil immersion (100×) in the microscope [18]. A commercially available staining reagent (K005 kit for ZN Acid Fast Stains, HIMEDIA, India) was used for staining of the stool samples. *C. parvum* was identified with its features: size (4–8 µm), oval or nearly spherical shape, pink color (Fig. 1), and *C. cayetanensis* oocysts were identified with their characteristic size (8–10 µm), round shape and red coloration (Fig. 2). Eggs of other protozoa and helminthes were also recorded.

### Water sampling and processing method

After collecting consent and the questionnaire, 100 ml of water samples were collected in labeled, clean, and sterile plastic container from each household. Soon after collection of water samples, the containers were kept in icebox, and were immediately transported to the Public Health Research Laboratory. All the samples were kept in freezer and within in 4 h of collection samples were centrifuged at 1500 rpm for 10 min. After centrifugation, both sediments and floated samples were taken and were suspended in 2.5% potassium dichromate solution, and the recovery of parasites of sporulation was noted by performing direct microscopy.

### Results

During the study period, stool samples were collected from 262 children and adults from 60 households in Thapathali slum in Kathmandu. Most of the participants (75.6%) were children less than 15 years of age. The proportion of male was 44.6%. Parasites were detected in 27.1% (71/262) of the stool samples, among them, protozoa and helminthes were detected in 21.4% (56/262) and 5.7% (15/262), respectively. *C. cayetanensis* and *C. parvum* were detected in 3.8% (10/262) and 1.5% (4/262), respectively. Characteristics of the study population and parasite detection are shown in Table 1. People whose used tanker water as source of drinking water had significantly higher proportions of protozoal and helmenthic parasites.

**Table 1:** Prevalence of protozoal and helmenthic parasitic infections among people living in a slum in Kathmandu

Characteristics	No of samples	Any parasite, no. (%)	Protozoal parasite, no. (%)	Helminthic parasite, no. (%)	*C. cayetanensis,* no. (%)	*C. parvum,* no. (%)
Age (years)						
<15	198	55 (27.8)	42 (21.2)	13 (6.6)	8 (4.0)	3 (1.5)
≥15	64	16 (25.0)	14 (21.9)	2 (3.1)	2 (3.1)	1 (1.6)
Sex						
Male	117	30 (25.6)	24 (20.5)	6 (5.1)	5 (4.3)	2 (1.4)
Female	145	41 (28.3)	32 (22.1)	9 (6.2)	5 (3.5)	2 (1.7)
Drinking water						
Tanker	102	58 (56.9)	43 (42.2)	15 (14.7)	6 (5.9)	3 (2.9)
Tap	89	10 (11.2)	10 (11.2)	0 (0.0)	3 (3.4)	1 (1.1)
Bottle	71	3 (4.2)	3 (4.2)	0 (0.0)	1 (1.4)	0 (0.0)
Education						
Illiterate	114	32 (28.1)	25 (21.9)	7 (6.1)	6 (5.3)	3 (2.6)
Literate	148	39 (26.4)	31 (21.0)	8 (5.4)	4 (2.7)	1 (0.7)
No. of members in household						
1–3	53	9 (17.0)	9 (17.0)	0 (0.0)	2 (3.8)	2 (3.8)
4–7	135	43 (31.9)	34 (25.2)	9 (6.7)	3 (2.2)	0 (0.0)
8–11	74	19 (25.7)	9 (17.0)	6 (8.1)	5 (6.8)	2 (2.7)
Occupation						
Student	163	45 (27.6)	35 (21.5)	10 (6.1)	8 (4.9)	3 (1.8)
Farmer	32	10 (31.3)	10 (31.3)	0 (0.0)	1 (3.1)	0 (0.0)
Factory worker	47	11 (23.4)	8 (17.0)	3 (6.4)	1 (2.1)	1 (2.1)
Others	20	5 (25.0)	3 (15.0)	2 (10.0)	0 (0.0)	0 (0.0)

There were ten different parasites detected in the stool samples (Table 2). *Giardia lamblia* was the commonest one (7.6%), followed by *Entamoeba coli* (4.2%), *Entamoeba histolytica* (3.8%). More than one parasite was detected in 10.3% (27/262) of the population.

**Table 2:** Parasites detected in stool samples of the people living in Thapathali slum, Kathmandu

Parasites	Prevalence, N = 262 (%)
*Giardia lamblia*	20 (7.6)
*Entamoeba coli*	11 (4.2)
*Entamoeba histolytica*	10 (3.8)
*Cyclospora cayetanensis*	10 (3.8)
*Ascaris lumbricoides*	8 (3.1)
*Cryptosporidium parvum*	4 (1.5)
*Hymenolepsis nana*	4 (1.5)
*Trichuris trichiura*	2 (0.8)
*Sarcosystis hominis*	1 (0.4)
*Hymenolepis diminuta*	1 (0.4)
Any one of parasites	71 (27.1)

### Discussion

This study has documented prevalence of *C. cayetanensis* and *C. parvum* infections among people living in slum area in Kathmandu, Nepal. Ten different parasites were detected in stool samples. The prevalence of any single parasite was higher among children age less than 15 years, and people whose used tanker water as a drinking source. One quarters of people living in Thapathali slum in Kathmandu found to be infected with intestinal protozoa and helminthes parasites.

Nepal is a small developing country located in South Asia where intestinal parasitosis is found to be highly prevalent [19]. In overall parasites of slum area, infection rate of *G. lamblia* (7.6%) was highest detected followed by *E. coli* (4.2%), *E. histolytica* (3.8%), *C. cayetanensis* (3.8%), *A*. *lumbricoides* (3.1%), *H. nana* (1.5%), *C. parvum* (1.5%), etc. In slum people, infection rate of protozoa is higher than helminthes parasites. These findings are similar to Garcia et al. [20]. Of them *G. lamblia* (33.1%) was most common followed by *E. histolytica* 20.7%*, T. trichiura* (8.3%) and others. Highest frequency of *G. lamblia* among the slum people might be associated with poor sanitary and their personnel hygiene. Furthermore, the cyst of *G. lamblia* is resistant to the normal level of chlorination of drinking water and thus easily is transmitted through drinking water [20].

*Cyclospora cayetanensis* and *C. parvum* are reported to cause repeated outbreaks of gastroenteritis in Nepal [21]. However, epidemiology of *Cyclospora* and *Cryptosporidium* in people and their clinical manifestation are not well characterized in Nepal [22, 23]. In this study, the coccidian parasites *C. cayetanensis* and *C. parvum* were detected 3.5% and 1.5%, respectively. A previous study in similar set up showed higher prevalence (8.3%) of *C. cayetanensis* among children [20]. In this study population, the prevalence was lower (4.0%) among children less than 15 years of age, the difference in prevalence might be due to various factors: water and hygiene conditions might be better in this slum area as the overall the prevalence of intestinal parasite was also lower (27.1% vs. 43.4%); another factor may be we confirmed the parasites by using the modified acid fast staining method which was not used in the previous study. To best of our knowledge, this study shows the detection of *C. parvum* in a slum area in Nepal for the first time. In a study among healthy school children, 3–14 years of age in Kathmandu valley, *C. parvum* was detected in 0.79% [22], whereas in this study the prevalence was 1.5% among children (Additional file 1).

We could not find significant differences in prevalence of *C. cayetanensis* and *C. parvum* among age groups and sexes. This may be due to small sample size. In previous studies in Nepal, *C. cayentanensis* tends to be more prevalent among females and among 2–5 years of age group of children [21]; *C. parvum* detected more in male than female children who were admitted to a hospital due to diarrhea [24]. In Mexico, elementary school age children (6–11 years old) and younger children (3–5 years old) were frequently infected with *Cyclospora* [25]. In Peru, in an endemic community, *Cyclospora* was present among children 1–9 years old [26]. It might be due to the fact that the coccidian parasites are opportunistic and infect both immune–competent and immunocompromised patients. However, it may not occur after infection [27], some amount of immunity may be present in adults who are exposed to the infection, as the infection is less prevalent in adults living in endemic areas [28]. Similarly, in previous study of *C. parvum*, it seems to have lower prevalence of 0.79% between 3 and 14 years of age of children in Kathmandu valley [22]. Although all the age groups of children is affected by the infection, a high prevalence of *C. parvum* infection (16.1%) was observed among 4–6 years old age group, attributed to overcrowding, poor hygiene especially among school going children [24]. Children of lower age groups are more susceptible to infection by *C. parvum* compared to higher age groups due to less developed immune system and poor personal hygiene [26].

One of the water sources, tanker water, seemed to be associated with a higher prevalence of intestinal parasites including the coccidian parasites. Tanker water was not treated water; the infection rate of *C. cayetanensis* was found higher than bottled water in another study too [23]. In Kathmandu valley, the source of tanker water might be polluted [22]. *Cyclospora* oocysts have been isolated from sewage, river and municipal pipe water [22, 29]. Similarly, prevalence C. *parvum* in untreated was detected most than bottle water among slum people which is similar to the findings of previous study [22]. It might be due to the supply of water that is not tested and treated properly and regularly, and might be in risk of contamination from sewage.

Coccidian parasites infections are common among people dwelling slum area in Kathmandu Valley. As *C. cayetanensis* and *C. parvum* are known to be associated with fecal–oral route of transmission, it is directly or indirectly due to consumption of the contaminated water. As chlorination is not enough to get rid of these parasites, we suggest boiling drinking water coming from the high-risk sources.

## Limitations

Due to limitation of our laboratory capacity, we could not perform the PCR and florescent microscopy for the conformation of the parasites.

## Additional files

**Additional file 1.** Study of *Cryptosporidium parvum* and *Cyclospora cayetanensis* infections among people living in Thapathali slum area, Kathmandu, Nepal.

**Additional file 2.** Dataset.

**Additional file 3.** Codebook of dataset of “Detection of *Cryptosporidium parvum* and *Cyclospora cayetanensis* infections among people living in a slum area in Kathmandu valley, Nepal”.

## Abbreviations

*C. cayetanensis*: *Cyclospora cayetanensis*
*C. parvum*: *Cryptosporidium parvum*
*G. lamblia*: *Giardia lamblia*
*E. histolytica*: *Entamoeba histolytica*
*A. lumbricoides*: *Ascaris lumbricoides*
*H. nana*: *Hymenolepsis nana*
*T. trichiura*: *Trichuris trichiura*
*S. hominis*: *Sarcosystis hominis*
*H. diminuta*: *Hymenolepsis diminuta*
*E. coli*: *Entamoeba coli*
No.: number
spp.: species
%: percentage
SICOST: Shi-Gan International College of Science and Technology
PCR: polymerase chain reaction

## References

[1] Quintero-betancourt W, Peele ER, Rose JB (2002). *Cryptosporidium parvum* and *Cyclospora cayetanensis*: a review of laboratory methods for detection of these waterborne parasites. J Microbiol Methd.

[2] Huang D, Chappell C, Okhuysen P (2009). *Cryptosporidiosis* in children. Semi Pediatric Infect Dis.

[3] Chappell CL, Okhuysen PC, Sterling CR, DuPont HL (1996). *Cryptosporidium parvum*: intensity of infection and oocyst excretion patterns in healthy volunteers. J Infect Dis.

[4] Ajjampur SSR, Gladstone BP, Selvapandian D, Muliyil JP, Ward H, Kang G (2007). Molecular and spatial epidemiology of *cryptosporidiosis* in children in a semi urban community in South India. J Clin Microbiol.

[5] Sherchand JB, Larsson S, Rans BJ (1992). The incidence of rotavirus and enteric adenovirus diarrhea in children attending the outpatient Department of Kanti Children’s Hospital and general Practitioner in Kathmandu Area. J Nepal Med Assoc.

[6] Shariff M, Deb M, Singh R, Singh K (2002). *Cryptosporidium* infection in children with diarrhoea of acute onset. J Trop Pediatr.

[7] Ortega YR, Sterling CR, Gilman RH, Cama VA, Diaz F (1993). *Cyclospora* species—a new protozoan pathogen of humans. N Engl J Med.

[8] Soave R (1996). *Cyclospora*: an overview. Clin Infect Dis.

[9] Hart AS, Ridinger MT, Soundarajan R (1990). Novel organism associated with diarrhoea in AIDS. Lancet.

[10] Bendal RP, Lucas S, Moody A (1993). Diarrhoea associated with cyanobacterium-like bodies: new coccidian enteritis of man. Lancet.

[11] Gascon J, Corachan M, Bombi JA, Valls MA, Bordes JM (1995). *Cyclospora* in patients with traveller’s diarrhoea. Scand J Infect Dis.

[12] Markus MB, Frean JA (1993). Occurrence of human *Cyclospora cayetanensis* infection in sub-Africa (Letter). S Afr Med J.

[13] Torres-Slimming PA, Mundaca CC, Moran M, Quispe J, Colina O, Bacon DJ, Lescano AG, Gilman RH, Blazes DL (2006). Outbreak of cyclosporiasis at a naval base in Lima, Peru. Am J Trop Med Hyg.

[14] Sherchand JB, Ohara H, Sherchand S, Cross JH, Shrestha MP (1997). Intestinal parasitic infections in rural areas of Southern Nepal. J Inst Med.

[15] Huang P, Weber J, Sosin DM (1995). The first reported outbreak of diarrheal illness associated with *Cyclospora* in the United States. Ann Intern Med.

[16] Rabold JG, Hoge CW, Shlim DR (1994). *Cyclospora* outbreak associated with chlorinated drinking water (letter). Lancet.

[17] Bhattachan B, Panta YB, Tiwari S, Thapa Magar D, Sherchand JB, Rai G, Rai SK (2015). Intestinal parasitic infection among school children in Chitwan District Of Nepal. J Inst Med.

[18] Garcia LS, Bruckner DA, Brewer TC, Miller NJ (1983). Techniques for the recovery and identification of *Cryptosporidium* oocysts from stool specimens. J Clin Microbiol.

[19] Rai SK, Hirai K, Abe A (2002). Intestinal parasitosis among school children in a rural hilly area of Dhading District, Nepal. Nepal Med Coll J.

[20] Thapa Magar D, Rai SK, Lekhak B, Rai KR (2011). Study of parasitic infection among children of Sukumbasi Basti in Kathmandu valley. Nepal Med Coll J.

[21] Sherchand JB, Sherchand JB, Cross JH (2007). An epidemiological study of *Cyclospora cayetanensis* in Nepalese people. J Inst Med.

[22] Bhandari D, Tandukar S, Sherchand S, Thapa P, Shah PK (2015). *Cryptosporidium* infection among the school children of Kathmandu Valley. J Inst Med.

[23] Bhandari D, Tandukar S, Parajuli H, Thapa P, Chaudhary P, Shrestha D (2015). Cyclospora infection among school children in Kathmandu, Nepal: prevalence and associated risk factor. Trop Med Health.

[24] Dhakal DN, Bc RK, Sherchand JB, Mishra PN (2004). *Cryptosporidium parvum*: an observational study in Kanti Children Hospital, Kathmandu, Nepal. J Nepal Health Res Counc.

[25] Orozco-Mosqueda GE, Martı´nez-Loya OA, Ortega YR (2014). *Cyclospora cayetanensis* in a Pediatric Hospital in Morelia, Mexico. Am J Trop Med Hyg.

[26] Bern C, Ortega Y, Checkley W, Roberts JM (2002). Epidemiologic differences between *Cyclosporiasis* and *Cryptosporidiosis* in Peruvian children. Emerg Infect Dis.

[27] Sifuentes-Osornio J, Porrascortes G, Bendall RP, Morales-Villarreal F (1995). *Cyclospora cayetanensis* infection in patients with and without AIDS: biliary disease as another clinical manifestation. Clin Dis.

[28] Ghimire TR, Mishra PN (2005). Intestinal parasites and haemoglobin concentration in the people of two different areas of Nepal. J Nepal Health Res Counc.

[29] Ghimire TR, Mishra PN, Sherchand JB (2005). The seasonal outbreaks of *Cyclospora* and *Cryptosporidium* in Kathmandu, Nepal. J Nepal Health Res Counc.

