# Supplementary material for: Detection of Cryptosporidium parvum and Cyclospora cayetanensis infections among people living in a slum area in Kathmandu valley, Nepal
Source: BMC Res Notes. 2017 Sep 7;10:464. doi: 10.1186/s13104-017-2779-2 (PMC5590164; doi:10.1186/s13104-017-2779-2)
Supplement: Supplementary file 1 — Additional file 1. Study of Cryptosporidium parvum and Cyclospora cayetanensis infections among people living in Thapathali slum area, Kathmandu, Nepal. [file 13104_2017_2779_MOESM1_ESM.pdf]

Study of *Cryptosporidium parvum* and *Cyclospora cayetanensis* infections among people living in Thapathali slum area, Kathmandu, Nepal

Study ID: [__][__][__]

Date Today: [D][D]-[M][M]-[Y][Y]

Name of the respondent: [...]

Mobile no of respondent: [...]

DEMOGRAPHIC DETAILS

1. Name: [...]

2. Sex: M [__] / F [__]

3. Age in Years: [__][__]

SOCIOECONOMIC STATUS

4. Occupation:
1=Students [__] 2=Farmer [__] 3=Factory worker [__]
4= Others [__]

5. Education:
Illiterate [__]
Literate [__]

6. Number of People in the household: [__]

7. Water source:
1=Tap [__] 2= Tanker [__] 3= Bottle [__]

LAB REPORT

8. **Protozoal parasites**:

Giardia lamblia [__]

Entamoeba coli [__]

Entamoeba histolytica [__]

Cyclospora cayetanensis [__]

Cryptosporidium parvum [__]

Sarcosystis hominis [__]

Isospora beili [__]

Edolimax nana [__]

Blastocystis hominis [__]

9. **Helminthic parasites:**

Ascaris lumbricoides [__]

Hymenolepsis nana [__]

Trichuris trichiura [__]

Hymenolepis diminuta [__]

Taenia spp [__]

Hookworm [__]
