# Supplementary material for: Detection of Cryptosporidium parvum and Cyclospora cayetanensis infections among people living in a slum area in Kathmandu valley, Nepal
Source: BMC Res Notes. 2017 Sep 7;10:464. doi: 10.1186/s13104-017-2779-2 (PMC5590164; doi:10.1186/s13104-017-2779-2)
Supplement: Supplementary file 3 — Additional file 3. Codebook of dataset of “Detection of Cryptosporidium parvum and Cyclospora cayetanensis infections among people living in a slum area in Kathmandu valley, Nepal”. [file 13104_2017_2779_MOESM3_ESM.pdf]

Codebook of dataset of "Detection of Cryptosporidium parvum and Cyclospora cayetanensis infections among people living in a slum area in Kathmandu valley, Nepal"

Variables	Labels
**age_groups**	**age_groups**

type: string (**str10**)

unique values: **2** missing "": **0/262**

tabulation:

Freq.	Value
**198**	**"<15 years"**
**64**	**">=15 years"**

warning: variable has embedded blanks

Text

**household_group**	**household_group**

type: string (**str12**)

unique values: **3** missing "": **0/262**

tabulation:

Freq.	Value
**53**	**"1-3 members"**
**135**	**"4-7 members"**
**74**	**"8-11 members"**

warning: variable has embedded blanks

**education**	**education**

type: string (**str10**)

unique values: **2** missing "": **0/262**

tabulation:

Freq.	Value
**114**	**"Illiterate"**
**148**	**"Literate"**

**water_source**	**water_source**

type: string (**str6**)

unique values: **3** missing "": **0/262**

tabulation:

Freq.	Value
**71**	**"bottle"**
**102**	**"tank"**

```
                              89  "tap"

--------------------------------------------------------------------------------
occupation                                                            occupation
--------------------------------------------------------------------------------

                  type:  string (str14)

         unique values:  4                        missing "":  0/262

           tabulation:  Freq.  Value
                            47  "factory_worker"
                            32  "farmer"
                            20  "others"
                           163  "student"

--------------------------------------------------------------------------------
no_house_members                                                no_house_members
--------------------------------------------------------------------------------

                  type:  numeric (byte)

                 range:  [2,11]                        units:  1
         unique values:  10                        missing .:  0/262

                  mean:   5.70229
             std. dev:   2.31998

          percentiles:        10%       25%       50%       75%       90%
                                3         4         5         8         9

--------------------------------------------------------------------------------
Entamoeba_coli                                                    Entamoeba_coli
--------------------------------------------------------------------------------

                  type:  string (str12)

         unique values:  2                        missing "":  0/262

           tabulation:  Freq.  Value
                            11  "detected"
                           251  "not detected"

              warning:  variable has embedded blanks

--------------------------------------------------------------------------------
Entamoeba_histolytica                                      Entamoeba_histolytica
--------------------------------------------------------------------------------

                  type:  string (str12)

         unique values:  2                        missing "":  0/262
```

tabulation: Freq. Value
10 **"detected"**
252 **"not detected"**

warning: variable has embedded blanks

Giardia_lamblia **Giardia_lamblia**

---

type: string (**str12**)

unique values: **2**   missing "": **0/262**

tabulation: Freq. Value
20 **"detected"**
242 **"not detected"**

warning: variable has embedded blanks

Cyclospora_cayetanensis **Cyclospora_cayetanensis**

---

type: string (**str12**)

unique values: **2**   missing "": **0/262**

tabulation: Freq. Value
10 **"detected"**
252 **"not detected"**

warning: variable has embedded blanks

Cryptosporidium_parvum **Cryptosporidium_parvum**

---

type: string (**str12**)

unique values: **2**   missing "": **0/262**

tabulation: Freq. Value
4 **"detected"**
258 **"not detected"**

warning: variable has embedded blanks

Hymenolepsis_nana **Hymenolepsis_nana**

---

type: string (**str12**)

```
       unique values:  2                        missing "":  0/262

          tabulation:  Freq.  Value
                           4  "detected"
                         258  "not detected"

             warning:  variable has embedded blanks

-------------------------------------------------------------------------------
Hymenolepis_diminuta                                       Hymenolepis_diminuta
-------------------------------------------------------------------------------

                type:  string (str12)

       unique values:  2                        missing "":  0/262

          tabulation:  Freq.  Value
                           1  "detected"
                         261  "not detected"

             warning:  variable has embedded blanks

-------------------------------------------------------------------------------
Trichuris_trichiura                                         Trichuris_trichiura
-------------------------------------------------------------------------------

                type:  string (str12)

       unique values:  2                        missing "":  0/262

          tabulation:  Freq.  Value
                           2  "detected"
                         260  "not detected"

             warning:  variable has embedded blanks

-------------------------------------------------------------------------------
Ascaris_lumbricoides                                       Ascaris_lumbricoides
-------------------------------------------------------------------------------

                type:  string (str12)

       unique values:  2                        missing "":  0/262

          tabulation:  Freq.  Value
                           8  "detected"
                         254  "not detected"

             warning:  variable has embedded blanks

-------------------------------------------------------------------------------
Sarcosystis_hominis                                         Sarcosystis_hominis
```

```
                  type:  string (str12)

         unique values:  2                        missing "":  0/262

            tabulation:  Freq.  Value
                             1  "detected"
                           261  "not detected"

               warning:  variable has embedded blanks
```

---

any_parasite **any_parasite**

```
                  type:  string (str12)

         unique values:  2                        missing "":  0/262

            tabulation:  Freq.  Value
                            71  "detected"
                           191  "not detected"

               warning:  variable has embedded blanks
```

---

protozoal_parasite **protozoal_parasite**

```
                  type:  string (str12)

         unique values:  2                        missing "":  0/262

            tabulation:  Freq.  Value
                            56  "detected"
                           206  "non detected"

               warning:  variable has embedded blanks
```

---

helmenthic_parasite **helmenthic_parasite**

```
                  type:  string (str12)

         unique values:  2                        missing "":  0/262

            tabulation:  Freq.  Value
                            15  "detected"
                           247  "non detected"

               warning:  variable has embedded blanks
```
